# Supplementary figures and images for: An induced pluripotent stem cell-based chemical genetic approach for studying spinal muscular atrophy
Source: Front Neurosci. 2026 Feb 4;19:1695359. doi: 10.3389/fnins.2025.1695359 (PMC12913379; doi:10.3389/fnins.2025.1695359)

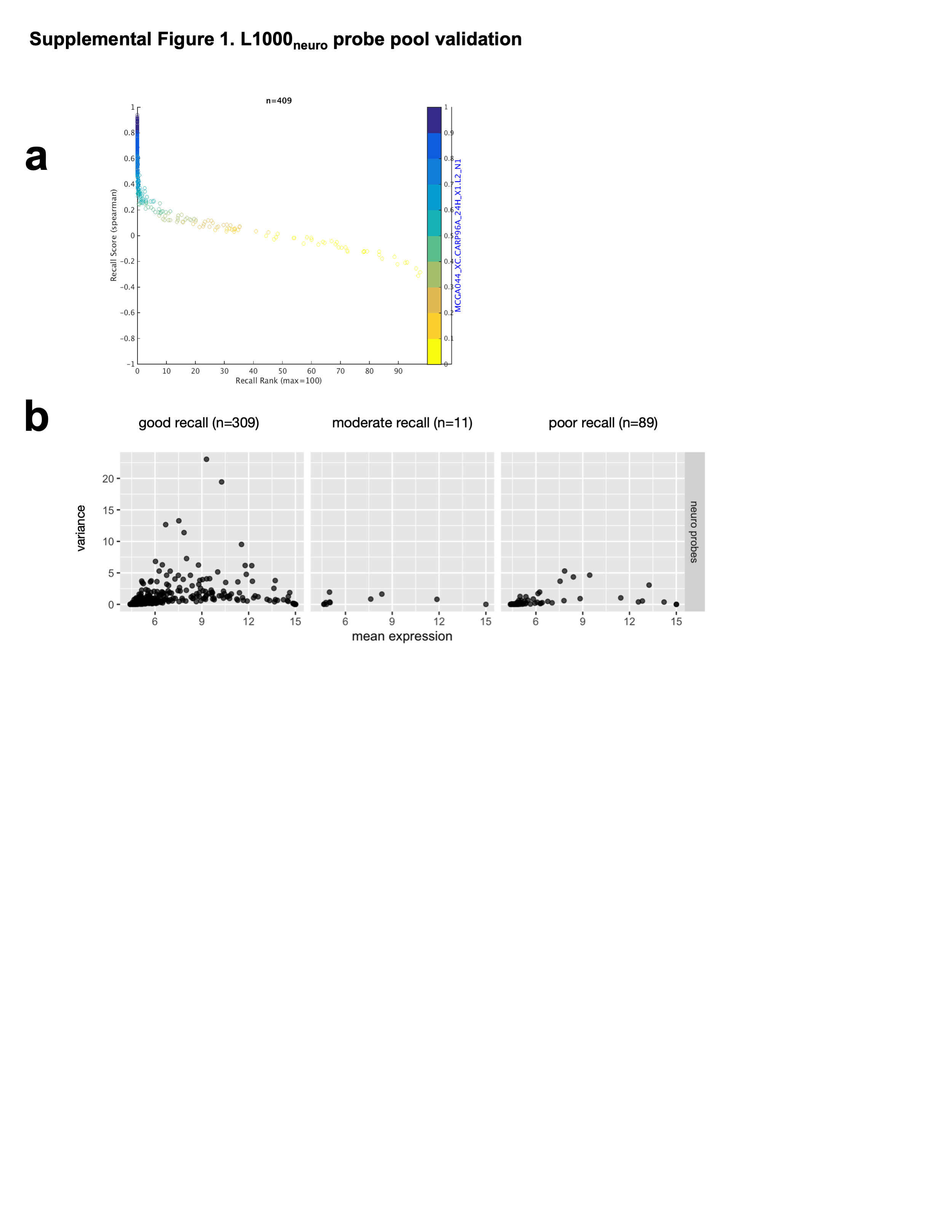

Supplement: SUPPLEMENTARY FIGURE 1 — L1000neuro probe pool validation. (a) Scatter plot depicting the Spearman correlation vs. the corresponding recall percentile rank for each of the 409 common genes. (b) Scatter plots of the 409 genes’ variance vs. mean expression, derived from the 96 CCLE cell lines’ log2 RPKM values, and stratified by whether the gene had good, moderate, or poor recall, corresponding to recall percentile ranks ≤5%, >5% & ≤10%, and >10%, respectively. [file Image_1.tiff]

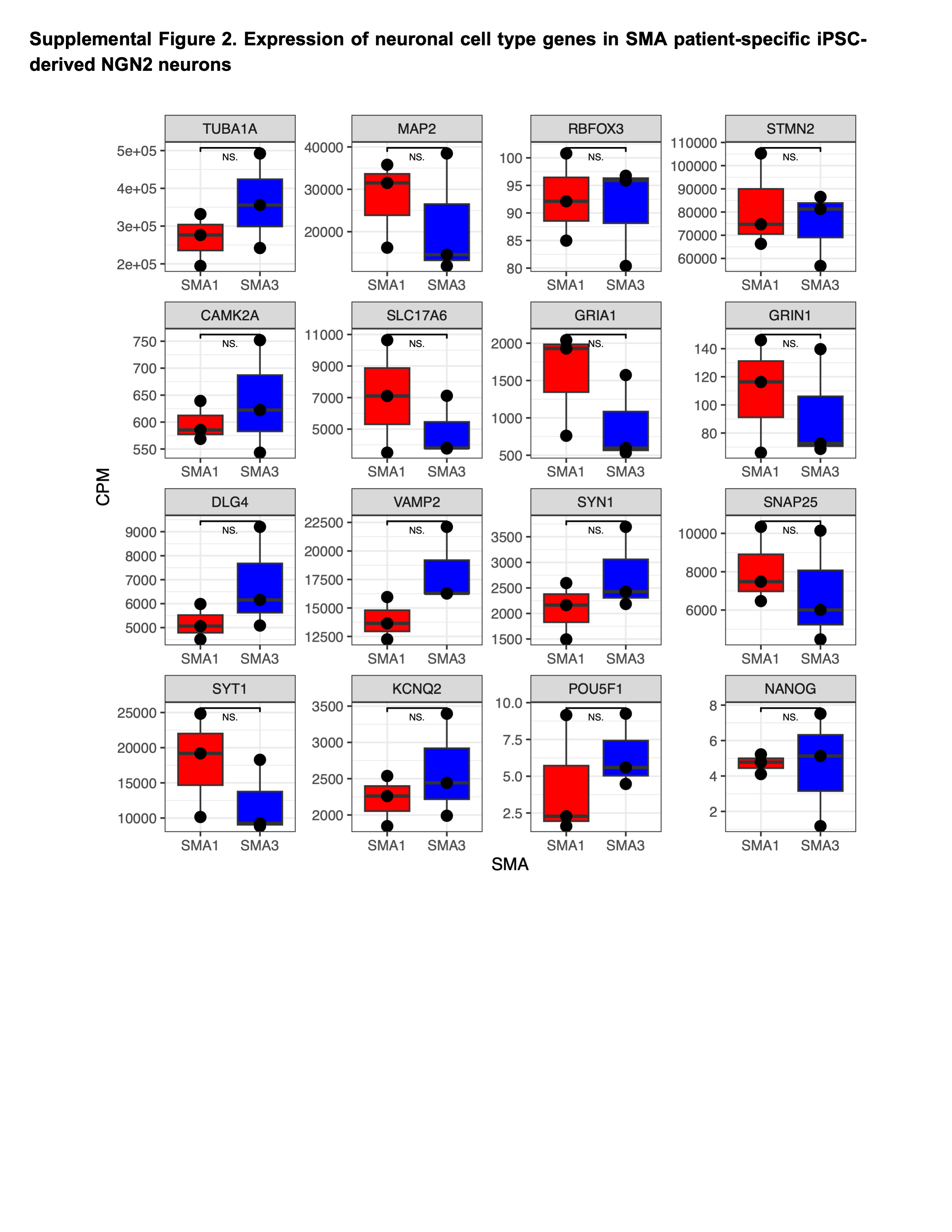

Supplement: SUPPLEMENTARY FIGURE 2 — Expression of neuronal cell type genes in SMA patient-specific iPSC-derived NGN2 neurons. NGN2 neurons from either SMA type exhibit high expression of pan-neuronal (TUBA1A, MAP2, RBFOX3, STMN2), glutamatergic (CAMK2A, SLC17A6, GRIA1, GRIN1), and synaptic/functional markers (DLG4, VAMP2, SYN1, SNAP25, SYT1, KCNQ2) with minimal expression of pluripotency genes (POU5F1, NANOG). No differences in expression were observed across SMA types (Wilcoxon Rank Sum test, p < 0.05). Dots depict individual cell lines; CPM, counts per million. [file Image_2.tiff]
